# Unilaterally Applied Resistance to Swing Leg Shows a Different Adaptation Pattern Compared to Split-Belt Treadmill in Patients with Stroke

**DOI:** 10.3390/brainsci13020264

**Published:** 2023-02-03

**Authors:** Nama Mizrachi, Simona Bar-Haim, Iuly Treger, Itshak Melzer

**Affiliations:** 1 Physical Therapy Department, Faculty of Health Sciences, Recanati School of Community Health Professions, Ben-Gurion University of the Negev, Beer-Sheva 84417, Israel; 2 The BGU Adi-Negev Translational Laboratory, Merhavim Regional Council, Ofakim 80300, Israel; 3 Rehabilitation Department, Soroka University Medical Center, Faculty of Health Sciences, Ben-Gurion University of the Negev, Beer-Sheva 84417, Israel

**Keywords:** post-stroke hemiparesis, motor adaptation, perturbation training

## Abstract

Persons with chronic stroke (PwCS) have a decreased ability to ambulate and walk independently. We aimed to investigate the differences between the motor adaptation process for two different perturbation methods: split-belt treadmill walking and unilaterally applied resistance to the swing leg during walking. Twenty-two PwCS undergo split-belt treadmill walking and unilaterally applied resistance to the swing leg during walking, each one week apart. The test included three phases: the baseline period, the early-adaptation period and the late-adaptation period, as well as the early-de-adaptation period and the late-de-adaptation period. The average step length, swing duration, double-limb support duration, and coefficient of variance (CV) of these parameters were measured. During the split-belt treadmill walking, PwCS showed an adaptation of double-limb support duration symmetry (*p* = 0.004), specifically a trend between baseline versus early-adaptation (*p* = 0.07) and an after-effect (late-adaptation compare to early-de-adaptation, *p* = 0.09). In unilaterally applied resistance to the swing leg during walking, PwCS showed lower swing phase duration CV, in the adaptation period (baseline compare to adaptation, *p* = 0.006), and a trend toward increased variability of gait in the de-adaptation period compare to the adaptation periods (*p* = 0.099). The rate of adaptation and de-adaptation were alike between the two perturbation methods. Our findings show that the learning process happening in the central nervous system of PwCS may be dependent on the nature of the perturbation (mechanical resistance vs. split-belt) and that PwCS are able to adapt to two types of errors.

## 1. Introduction

Stroke is a main reason of disability and mortality [1]. The death rate from stroke was 76.8 per 100,000 for persons aged 65–74 years, 256.0 per 100,000 for persons aged 75–84 years and 984.3 per 100,000 persons aged ≥85 years [1]. Stroke involves of sensory and motor impairments such as muscle weakness, impaired postural control, spasticity, and somatosensory deficit affecting gait [2,3]. Also, the degree of disability affected the employability of patients with chronic stroke (PwCS). It was found that motor deficits, aphasia, mental status disorders, and National Institutes of Health Stroke Scale score at admission and the modified Rankin Scale at 6-month follow-up, were independently associated with employment after stroke [4]. In this study 20–42% PwCS could not go back to work half a year after hospital discharge [4,5,6]. One of the major concerns of PwCS is the capability to walk independently [7]. Improved gait in people with stroke is associated with improvement in cardio-vascular function, and in participation, and reduction in stroke recurrence [8]. Therefore, improving walking capability is an important goal during the rehabilitation period of PwCS. 

While temporal and spatial parameters of gait are symmetrical among healthy adults [9,10], gait among people with stroke is characterized by a-symmetry, poor motor control, and lower weight bearing on the affected limb [11]. Gait impairments in PwCS characterized by a decreased gait speed and a reduction in the number of steps per minute, a-symmetry of step length [11,12,13], reduced gait efficiency [14], and a reduction in dynamic balance [15]; hence, a lower ability to ambulate safely, i.e., inability to cross a street [16]. Calculations of gait symmetry often use swing-time duration, stance-time duration, or an intra-limb ratio of swing-time duration to stance-time duration. Commonly, hemiparetic gait is characterized by a longer affected leg swing time and/or a longer non-paretic leg stance time compare to the unaffected limb [17]. Spatial symmetry calculations using step length reported that 55.5% of a group of hemiparetic PwCS had temporal gait asymmetry, while only 33% of PwCS show spatial gait asymmetry [9]. Since PwCS are heterogenic, some may demonstrate longer affected step, while others demonstrate shorther affected step [13]. Beaman et al. [18] reported that 39% of PwCS walked with longer affected leg steps at their comfortable walking speed, 15% of PwCS walked with shorter affected limb step length, and 45% of PwCS walked with symmetric step length. 

Various rehabilitation interventions have employed motor learning laws, such as motor adaptation that targeted specific gait disorders [19,20,21,22]. Savin et al. [23] found that gait asymmetry among PwCS with hemiparesis could be temporarily altered t after exposure to a perturbation during the swing phase. Motor adaptation is defined as a practice-dependent changes or modification of a well-learned motor skill and movement pattern caused by a sensorimotor perturbation or an error feedback [24]. The process of adaptation happens over a stage of trial-and-error training in response to a new demanding task [24,25]. Motor adaptation is an important factor of motor learning and considered as indication for short-time motor learning. In case a new movement pattern is fully adapted, storing the new pattern in the central nervous system is exhibited through after-effect, i.e., opposite movement errors to those seen during the beginning of the adaptation period. After-effects show that the central nervous system updated a novel feed-forward motor command [26,27]. In order to return to the earlier movement performance, the participant have to de-adapt during an episode of continuous repetitions without the perturbation [28]. 

Studies showed that asymmetric gait in PwCS is initially increased after exposure to a constant perturbation, then resulting after-effects that can improve gait symmetry temporarily. Helm et al. [16] used a split-belt practice paradigm i.e., the participant walked while the two belts are moving at a similar velocity, or with the two treadmills belts moving at different velocities. During the training, paradigm inter-limb parameters, for example, doubled limb support time and step length, and changed gradually, with significant adaptations following the return to normal treadmill walking [16]. Savin et al. [23,29] examined participants while walking on a treadmill with a cable that fastened above the ankle joint, having the step length to become shorter. The other end of the cable was attached to a weight equivalent l to 1.25 percent of the subject’s weight, provided resistance to forward movement of that lower limb during the swing phase [23,29]. This resistance i.e., perturbation, caused a substantial step length symmetry after-effect. In addition, the step length symmetry after-effects generalized to over ground gait, indicating that PwCS and hemiparesis, can alter their motor systems output through learning mechanisms based on trial and error [23]. 

In the present study, we aimed to explore the differences between the motor adaptation processes between two different perturbation methods: split-belt treadmill walking, i.e., velocity perturbation versus unilaterally applied resistance to the swing leg during treadmill walking, i.e., mechanical perturbations. We hypothesized that both perturbations would show an equivalent motor adaptation process.

## 2. Materials and Methods

### 2.1. Participants

In an explorative laboratory study, we recruited 22 PwCS who had unilateral stroke 3–19 months before conducting the study. The inclusion criteria were: a history of unilateral hemiparesis, ability to walk independently (maybe with a cane, but not with a walker), ability to follow instructions, and providing medical permission from their physician. The exclusion criteria were: Mini-Mental Examination Score <24, people with lower-limb amputation, Meniere’s disease or other vestibular impairments, respiratory disorders, cardiovascular disorders not allowing walking, usage of a pacemaker, and under active treatment of cancer or metastases. The study was approved by the Helsinki Ethics Committee of Soroka Medical Center (Unique Protocol ID: SOR-0411-15-ctil) and all participants signed a written informed consent.

### 2.2. Assessment Protocol

PwCS enrolled in the study walked on a motorized treadmill twice, one week apart to compare rates of adaptation and de-adaptation of the paretic leg in response to velocity perturbation (i.e., split-belt treadmill walking) and unilaterally applied resistance to the swing leg during walking given to the paretic leg using the “Just walk” device. During these two experiments, the PwCS walked on the same motorized treadmill that has two split belts and an inserted force plate under the treadmill (ForceLink, Clemborg, The Netherlands) [30]. The test included three phases: baseline, adaptation, and de-adaptation [31]. During the first experiment, participants walked on the split belt treadmill with the belts moving at a similar velocity (“tied” configuration) or at different velocities (“split-belt” configuration) [21]. The split belts velocity was determined for each participant out of three different protocols: (1) 0.23 m/s and 0.4 m/s, (2) 0.4 m/s and 0.7 m/s, or (3) 0.6 m/s and 1.05 m/s; based on their over ground gait speed. For all of PwCS who participated in the study, the leg assigned to the fast belt was always the paretic leg with the purpose of exaggerating step length asymmetry during split-belt adaptation, as suggested by previous studies [32,33]. During the baseline period, participants walked belts moving at a similar velocity (“tied” configuration) at a slow walking speed for 2 min, then at a high walking speed for another 2 min, and finally at low walking speed again for 2 min. During the adaptation period, the treadmill belts were split (one belt faster than the other belt) for 10 min. In the de-adaptation period, participants walked at a slow walking speed for 5 min. The treadmill belts were stopped between each period for 10 s. 

During the second experiment, the “Just walk” device [34] was used to evoke external resistance for forward movement of the paretic leg during the swing phase of gait. The “Just walk” portable system is a device fixed in the belt placed around the participant’s waist. The device provides constant linear, and modifiable magnetic force converted into kinetic energy. A tension cable in the device is attached to the participant’s feet and ankle by adjustable foot bands. There are four resistance magnitudes, equivalent to 300–1, 400 g, created by magnets and metal discs, providing resistance to the swinging leg through the tension cable proportional to the movement speed. In the current study, the tension cable of the “Just walk” was linked to the posterior part of the ankle of the paretic leg, providing resistance to the forward movement of during the swing phase, and compression forces of the joints of the lower limb. During all periods of the second experiment, participants walked on the split belt treadmill with the belts moving at the similar velocity set to their preferred walking speed, similar to the first experiment. The participant’s walked during the bassline period for 5 min without resistance to their swinging leg. During the adaptation period, participants walked for 10 min, while the tension cable of the device was linked to the ankle of the paretic leg, providing forward resistance during the swing phase. During de-adaptation period, the cord was de-attached, and participants walked for 5 min without perturbation. The treadmill belts were stopped between each phase to attach and de-attach the cord. 

In both experiments, a safety harness system was worn to prevent a fall, but not to support body weight or limit limb joints’ range of motion during walking. 

### 2.3. Data Analysis

Custom software written in C# (Microsoft Visual Studio, Microsoft Ltd., Redmond, WA, USA) was used for controlling the velocities of the split belts and the breaks durations in the experiment. Ground reaction forces (GRF) and center of pressure (CoP) were sampled and recorded with Gaitfors software (ForceLink. 124. BV, Clemborg, The Netherlands). The system recorded the forces with one-dimensional force sensors from a single large (160 × 800 mm) force plate fixed in the split belt treadmill. Force data was collected at 500 Hz. The software has the ability to determine kinematic data and the gait events such as initial contact, toe-off and mid-stance for each leg independently. 

**Figure 1:**
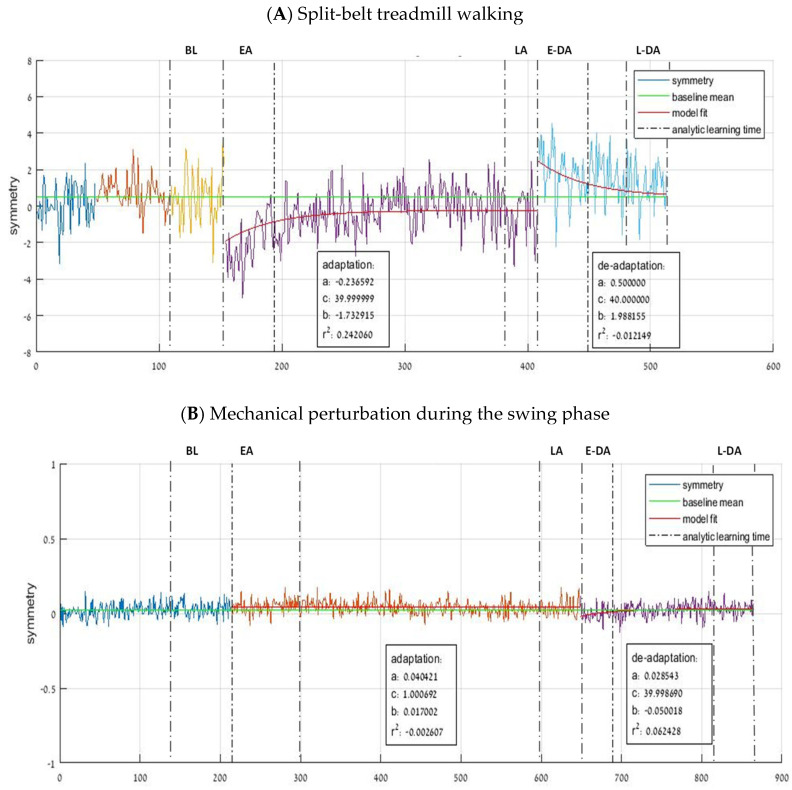
An example of kinematic adaptation data. Ground reaction forces (GRFs) and center of pressure (CoP) were sampled and recorded with Gaitfors software (ForceLink. 124. BV, Clemborg, The Netherlands). Here, we present adaptation and de-adaptation periods during (**A**) split-belt treadmill walking and (**B**) mechanical perturbation during swing phase, using an exponential decay function with the form of $y=a+(b\times e^{-\frac{t}{c}})$. The vertical lines represent the baseline period, the early-adaptation period, late-adaptation period, early de-adaptation period, and late de-adaptation period (See text for further details). Note: a—the final value that the exponential decay function approaches. b—the magnitude of adaptation or de-adaptation required from the first trial value to the value a. t—the stride number. c—the number of strides it will take to obtain $1-e^{-1}$ or approximately two-thirds of the adaptation or de-adaptation. r^2^—fitting of the curve. (**A**) split-belt treadmill walking, (**B**) mechanical perturbation during the swing phase. Note: x-axis is the time in milliseconds and y-axis is the symmetry index (SI, see text for elaboration). Perfect symmetry will result in an SI value of zero, a positive value indicates that the variable on the right side is less than the one measured on the left side and a negative value indicates that the variable on the left side is less than the one measured on the right side. Abbreviations: baseline period (BL); the early-adaptation period (EA); late-adaptation period (LA); early de-adaptation period (E-DA); and late de-adaptation period (L-DA).

Analysis was based on measurements of the vertical GRF profiles that -PwCS produced during different phases of gait. The GRFs were calculated in single-limb and in double-limb support, and then were normalized for each participant’s weight. The symmetry and variability of the following parameters were calculated from the kinematic data that were extracted by the system during the gait cycle events: (1) Step length—defined as the forward distance in millimeters between initial-contact (IC) of one foot to IC followed by the other foot, (2) Swing duration— defined as the duration in seconds between toe-off (TO) of one foot to IC of the same foot, and (3) Double-support duration— defined as the duration in seconds between IC of one foot to the next TO of the other foot. Symmetry was quantified with a symmetry index (*SI*): $SI=\frac{X_{L}-X_{R}}{X_{L}+X_{R}}$, where *L* is the left foot, *R* is the right foot, and *X* represents the variable of interest (i.e., step length). Perfect symmetry will result in an *SI* value of zero, aa positive value indicates that the variable on the right side is less than the one measured on the left side and a negative value indicates that the variable on the left side is less than the one measured on the right side [23,35]. In addition, the coefficient of variance (CV) with the form of $\frac{Standard\;deviation}{mean}\times 100\;$ was calculated for the SIs of spatial and temporal parameters. To determine adaptation and de-adaptation rates, an exponential decay function was then fit to each participant’s data in the form of $y=a+(b\times e^{-\frac{t}{c}})$, (Figure 1), where a is the final value that the exponential decay function approaches, i.e., the plateau reached at the end of adaptation or de-adaptation, *b* is the magnitude of adaptation or de-adaptation required from the first trial value to the value *a*, *t* is the stride number, and *c* is the decay constant or the rate at which adaptation or de-adaptation occurs. In this paradigm, c is the number of strides it will take to obtain $1-e^{-1}$ or approximately two-thirds of the adaptation or de-adaptation. In order to ensure a logical and reasonable fit, we broadly constrained to vary between −0.25 and +0.25, *b* was set to equal the difference between the first and final adaptation or de-adaptation symmetry values on a participant by participant basis, and *c* to vary between 1 and 40 [23,36].

### 2.4. Statistical Analysis

The effects of the two types of perturbation on the mean dependent variables (gait symmetry parameters) were analyzed by SPSS 17 software (Chicago, IL). To compare the different periods of the experiment, for each participant, we first calculated f the average value (±SD) of all parameters in (1) the last 30 gait cycles during the baseline period, (2) the average of the first five cycles of gait in the adaptation period (early-adaptation period), (3) the last five cycles of gait in the adaptation period (late-adaptation period), (4) the average of the first five cycles of gait in the post-adaptation period (early-de-adaptation period), and (5) the last five cycles of gait in the post-adaptation period (late-de-adaptation period). (see Figure 1A,B). Since the dependent variables in both experiments were not normally distributed (i.e., Shapiro-Wilk’s statistic), a non-parametric Friedman test wad used to compare between the two different perturbations (split-belt treadmill walking vs. unilaterally applied resistance to swing leg during walking) during the five different periods for kinetic parameters (baseline, early-adaptation, late-adaptation, early-de-adaptation, and late-de-adaptation). Wilcoxon’s signed rank tests were used to compare the rates of adaptation and de-adaptation between the two experiments i.e., split-belt treadmill adaptation versus unilaterally applied resistance to swing leg adaptation. The significance level was set to 0.05. 

## 3. Results

Twenty-two PwCS (mean age 59.3 ± 11.7 years), 3–19 months after stroke (average 26.9 ± 43.29 after stroke), participated in the study. Fourteen with right hemiparesis and 8 left hemiparesis. Eighteen PwCS were male and four females, 18 PwCS were cane users, and four were none-cane users.

### 3.1. Gait Symmetry Motor Adaptation and De-adaptation during Split-Belt Treadmill Walking and Unilaterally Applied Resistance to Swing Leg during Walking 

During split-belt treadmill walking, a significant effect was found in double-support duration symmetry (*p* = 0.004, Table 1). Post-hoc analysis showed a trend towards significance in double-support duration symmetry between the baseline versus the early-adaptation phases (0.008 ± 0.07 vs.−0.047 ± 0.13, *p* = 0.07), and between the late-adaptation versus the early de-adaptation phases (0.006 ± 0.08 vs. 0.07 ± 0.1, *p* = 0.09).

In regard to unilaterally applied resistance to the swing leg during walking, a significant difference was found for the swing phase duration CV between the different phases of adaptation (*p* = 0.002, Table 2). Post-hoc analysis revealed a significant decrease in the swing phase duration CV between the baseline and adaptation phases (142.79 ± 199.52 vs. 63.59 ± 43.82, *p* = 0.006) and a trend toward significance between the adaptation and de-adaptation phases (63.59 ± 43.82 vs. 93.03 ± 72.36, *p* = 0.099). A significant difference was also found for the double-support duration CV between all phases (*p* = 0.016, Table 2). However, post-hoc analysis revealed only a trend toward significance in the double-support duration CV between the adaptation and de-adaptation phases (274.33 ± 563.68 vs. 308.7 ± 397.52, *p* = 0.084), while there were no significant differences between de-adaptation and baseline phases (*p* = 0.758, Table 2). During the split-belt treadmill walking, a trend toward significance was found for step length CV and swing phase duration CV (*p* = 0.062 and *p* = 0.075, respectively).

### 3.2. Rates of Adaptation and De-adaptation during Split-Belt Treadmill Walking and Unilaterally Applied Resistance to Swing Leg during Walking

Table 3 shows that there were no significant differences between split-belt treadmill walking and unilaterally applied resistance to the swing leg during walking in the rate of adaptation or the rate of de-adaptation for step length symmetry (*p* = 0.705, *p* = 0.432), swing duration symmetry (*p* = 0.794, *p* = 0.553), or double-support duration symmetry (*p* = 0.185, *p* = 0.872). 

## 4. Discussion

PwCS in our study have the ability to alter their motor output in reaction to two different perturbation types during walking (i.e., split-belt treadmill walking and unilaterally applied resistance to a forward movement of the paretic leg during the swing phase of the gait cycle). Gait symmetry parameters were more influenced in response to split-belt treadmill walking, whereas unilaterally applied resistance to the paretic leg influenced the gait variability (i.e., CV). To our knowledge, only a few studies examined the differences in adaptation to different types of perturbation, but these focused on reaching movement of the arm [37,38,39].

We found an adaptation of double-support duration symmetry in PwCS during the split-belt treadmill walking (*p* = 0.004), specifically between the baseline vs. early-adaptation periods (*p* = 0.07) and an after-effect (late-adaptation vs. early-de-adaptation, *p* = 0.09). The above parameters, were not influenced by unilaterally applied resistance to the paretic leg. In unilaterally applied resistance to the swing leg during walking, PwCS demonstrated a different adaptation pattern. They showed a less variable gait during the adaptation period i.e., swing phase duration CV, during the adaptation period (baseline vs. adaptation, *p* = 0.006), as well as a trend toward increased variability of gait in the de-adaptation period versus the adaptation period (*p* = 0.099). During split-treadmill walking, however, PwCS showed only a trend toward significance in these parameters (step length CV and swing duration CV). Even so, the degree of adaptation and de-adaptation were identical between split-belt treadmill walking and unilaterally applied resistance to the paretic leg. These results are in partial agreement with Wei et al. [38] who found that during trial-by-trial motor adaptation with perturbations that were applied randomly, although the central nervous system show learning abilities to oppose perturbations, it does so in a way that is independent of the specific character of the perturbation. They suggested that the underlying motor learning process occurring in the central nervous system in PwCS as a feed-forward control motor output command in anticipation of perturbations may not be dependent of the nature of the perturbation (in our study, concerning split-belt treadmill perturbation vs. unilaterally applied resistance to the swing leg during walking). 

The results of our study differ from past studies that studied the effects of mechanical perturbations resisting the affected leg of PwCS during its swing phase on step length symmetry [23,40,41]. While we found no adaptation nor de-adaptation of step length symmetry, Savin et al. [39] found that external perturbation caused by a pulley system created an Instant change in step length symmetry in the early adaptation period, while in the late adaptation period, step length symmetry altered back towards similar to baseline values. When the external perturbation was eliminated, a negative after-effect was shown, and that the step length symmetry reverted to baseline values by the end of the de-adaptation [40]. Using the same pulley system to induce the perturbation, Savin et al. [23] demonstrated that step length adaptation was generalized to over-ground walking. The perturbation in their study [23,29,40] caused the step length symmetry index to increase in early-adaptation period versus the baseline period, while in late-adaptation period, the step length symmetry index altered towards (but remained above) baseline values. During generalization to over-ground walking, a negative after-effect occurred, presenting a more symmetrical step length. After-effect resulted in temporarily walking with step length symmetry over-ground compared with baseline, when the generalization ends, however, the step length symmetry index returned to baseline [23]. Yen et al. [41] studies how PwCS with a shorter step length of the affected leg responded to unilateral resistance applied to the swinging leg during treadmill walking. The symmetry of step length had a minor alteration during the early-adaptation (i.e., the symmetry index deviated a bit more from 0), and became more asymmetrical compared to the baseline period during the late-adaptation period. When the external applied resistance was removed during the de-adaptation period, the step length improved and became more symmetrical compared to the baseline period, and retained for nearly 10 steps [35]. We found significant difference in the gait variability parameters, but we did not find differences in the step length symmetry. One possible explanation for the differences between our and the previous results is the different vector of the external mechanical force applied to the paretic leg in our study. While, the systems used by the previous studies generated a large horizontal force vector to the paretic leg, the portable device used in our study evoked mechanical resistance that induced a smaller horizontal force vector, and larger vertical force vector, applying a compression forces to the paretic leg lower limb joints during its swing phase. The small horizontal vector applied to the paretic leg apparently results in insufficient resistance to the forward movement of the paretic leg during its swing phase. Another possible explanation for the differences between our results and previous ones [32,39] is that in the other investigations the mechanical perturbation was applied to the leg with the shorter step. In our study however, the mechanical perturbation was applied always to the paretic leg regardless of the direction of the initial a-symmetry (i.e., which leg had the shorter step). Savin et al. [40] found that applying external resistance to the swinging paretic leg of PwCS with a longer paretic leg step length initially decreased that step length during the early adaptation period (due to the additional force in the opposite direction of the swing), and resulted in an increase in step length with the paretic leg during the de-adaptation period as the participant adapted to the additional resistance. This would result in a decrease in step length symmetry during the de-adaptation period for these participants. Thus, it was suggested that applying swing resistance to the paretic leg during treadmill walking may or may not correct step length asymmetry of PwCS, depending on the direction of the step length symmetry at the baseline period [40]. In addition, Malone and Bastian [32] demonstrated that a similar split-belt adaptation led to other after-effects depending on the initial asymmetry of the PwCS. Walking asymmetry can be deteriorate or improved in de-adaptation period depending on a patient’s asymmetry and which limb is on the “slower” belt of the split-belt treadmill. For example, if PwCS take a smaller step with their paretic limb, the training should be with their non-paretic limb on the slower split-belt treadmill [32]. 

Thus, to further examine the effect of initial asymmetry, we divided the PwCS into two groups: those with a shorter paretic step (n = 12) and those with a longer paretic step (n = 10), with the main interest on the PwCS with the shorter paretic step. Results revealed no significant differences in gait symmetry parameters (i.e., step length symmetry and swing duration symmetry) through the testing periods in PwCS with the shorter paretic step.

In our study, we also found that although PwCS did not show changes in gait symmetry parameters during unilaterally applied resistance to the paretic leg during the swing phase, they did demonstrate a less variable gait, as shown by swing phase duration CV during the adaptation period compared to the baseline period, as well as a trend toward less variable gait during the adaptation period compared to the de-adaptation period. A past study [42] indicated a marked increase in the stride-to-stride variability in the gait of older adults who reported previous falls, suggesting that the measure of gait variability provides a quantitative assessment of gait instability that predisposes people to falls. Thus, our results show that PwCS were more stable during unilaterally applied resistance to the paretic leg, i.e., a decrease in swing phase duration variability during the adaptation period compared to the baseline. One way to explain these findings is the reduced proprioception in a majority (89%) of people with PwCS [43] since proprioceptive feedback is crucial in adapting and learning a new dynamic model [44]. Thus, we assume that due to a larger vertical compression force to the lower limb joints a more significant proprioceptive stimulus to the paretic lower limb joints was provided, PwCS in our study were able to adapt and learn the kinematics of walking and show more stable gait (i.e., reduced CV). It should be measured whether these improvements in gait variability were retained after a longer period of walking and treatment time, and reduce falls, highlighting the clinical relevance of using this method of training in gait rehabilitation programs for PwCS.

Our study has several limitations, mainly the small number of participants and their heterogenic character (i.e., shorter paretic step vs. longer paretic step). This may have influenced the results and made them indistinct to PwCS with shorter and longer paretic step. In addition, we did not measure sensory system impairment and proprioception in both lower limbs, specifically the affected lower limb. This may have hindered our ability to find whether the ability to adapt to the unilateral resistance is related to proprioceptive input.

## 5. Conclusions

PwCS in the present study demonstrated improved swing phase duration CV (i.e., single support phase), in the adaptation period when external resistance was applied to the paretic leg while walking. This may be caused by the vertical compression force provided by the ’just walk" device triggering a more significant proprioceptive stimuli to the lower limb joints specifically during the swing phase. During the split-belt treadmill walking however, PwCS showed specific adaptation of double-limb support duration symmetry (i.e., during double-support phase). This suggests that the underlying motor learning process happening in the central nervous system of PwCS as a feed-forward control command is specific and may be dependent on the nature of the perturbation (mechanical resistance to the swinging limb vs. split-belt perturbation to the standing limb) and that the accurate direction of force applied to the paretic leg while walking is an important factor in the ability to adapt. Further investigation is needed to better understand the adaptive mechanism to mechanical perturbation and its clinical implications and relevance. 

## Figures and Tables

**Table 1:** Gait symmetry during gait on (1) split-belt treadmill, and (2) unilaterally applied resistance to swing leg. Values presented as mean ± SD.

1. Split-Belt Treadmill	Baseline	Early-Adaptation	Late-Adaptation	Early De-adaptation	Late De-adaptation	Friedman Test	Post Hoc
Step length symmetry	−0.031 ± 0.10	−0.049 ± 0.17	−0.067 ± 0.20	−0.026 ± 0.21	−0.047 ± 0.12	χ^2^ = 1.38 *p* = 0.84	
Swing duration symmetry	−0.047 ± 0.18	−0.096 ± 0.24	−0.067 ± 0.19	−0.024 ± 0.19	−0.05 ± 0.19	χ^2^ = 4.72 *p* = 0.31	
Double-support duration symmetry	0.008 ± 0.07	−0.047 ± 0.13	0.006 ± 0.08	0.07 ± 0.10	0.028 ± 0.099	χ^2^ = 6.74 *p* = 0.004	Baseline vs. early de-adaptation, *p* = 0.07
							Early de-adaptation vs. late de-adaptation, *p* = 0.12
							Late-adaptation vs. early de-adaptation, *p* = 0.09
							Early de-adaptation vs. late de-adaptation, *p* = 0.22
							Late de-adaptation vs. baseline, *p* = 0.79
2. Unilaterally applied resistance to swing leg							
Step length symmetry	−0.01 ± 0.09	0.012 ± 0.14	−0.016 ± 0.10	−0.004 ± 0.05	−0.02 ± 0.10	χ^2^ = 3.67 *p* = 0.452	
Swing duration symmetry	−0.039 ± 0.16	−0.041 ± 0.17	−0.038 ± 0.19	−0.04 ± 0.18	−0.048 ± 0.17	χ^2^ = 1.6 *p* = 0.809	
Double-support duration symmetry	0.022 ± 0.07	0.05 ± 0.08	0.03 ± 0.09	0.019 ± 0.08	0.035 ± 0.08	χ^2^ = 0.36 *p* = 0.773	

Note: we used the Friedman test to compare between the testing periods.

**Table 2:** Gait variability (coefficient of variance, CV) during (1) split-belt treadmill walking, and (2) unilaterally applied resistance to swing leg, using the Friedman test through testing periods. Values are presented as mean ± SD.

1. Split-Belt Treadmill	Baseline	Adaptation	De-adaptation	Friedman Test	Post Hoc
Step length CV	724.0 ± 1693.9	183.6 ± 286.1	210.8 ± 163.2	χ^2^ = 5.52 *p* = 0.062	
Swing duration CV	250.7 ± 350.7	329.7 ± 117.4	210.2 ± 401.4	χ^2^ = 5.18 *p* = 0.075	
Double-support duration CV	755.6 ± 216.3	233.6 ± 235.3	256.2 ± 299.9	χ^2^ = 1.18 *p* = 0.55	
2. Unilaterally applied resistance to swing leg					
Step length CV	209.2 ± 248.0	278.57 ± 451.72	299.0 ± 609.7	χ^2^ = 3.273 *p* = 0.195	
Swing phase duration CV	142.8 ± 199.5	63.59 ± 43.82	93.0 ± 72.4	χ^2^ = 12.091 *p* = 0.002	Baseline vs. adaptation: *p* = 0.006 Adaptation vs. de-adaptation, *p* = 0.099
Double-support duration CV	256.4 ± 264.8	274.33 ± 563.7	308.7 ± 397.5	χ^2^ = 8.273 *p* = 0.016	Baseline vs. adaptation: *p* = 0.15
					Adaptation vs. de-adaptation: *p* = 0.084
					de-adaptation vs. baseline: *p* = 0.758

**Table 3:** Rates of adaptation and de-adaptation during walking on the split-belt treadmill vs. unilaterally applied resistance to swing leg during walking.

Adaptation	Learning Rate (Mean ± SD)		z	*p*-Value	De-Adaptation	Learning Rate (Mean ± SD)		z	*p*-Value
	Split-Belt	Unilaterally Applied Resistance to Swing Leg				Split-Belt	Unilaterally Applied Resistance to Swing Leg		
Step length	18.4 ± 18.2	18.9 ± 18.1	−0.379	0.705	Step length	24.8 ± 16.1	21.2 ± 17.6	−0.785	0.432
Swing duration	18.3 ± 17.7	16.7 ± 16.7	−0.262	0.794	Swing duration	23.9 ± 17.1	22 ± 17.6	−0.593	0.553
Double-support duration	27.3 ± 15.9	20.3 ± 16.2	−1.326	0.185	Double-support duration	22.0 ± 17.2	22.3 ± 17.9	−0.161	0.872

## Data Availability

Not applicable.
